# Low motional impedance distributed Lamé mode resonators for high frequency timing applications

**DOI:** 10.1038/s41378-020-0157-z

**Published:** 2020-06-15

**Authors:** Anosh Daruwalla, Haoran Wen, Chang-Shun Liu, Farrokh Ayazi

**Affiliations:** School of Electrical and Computer Engineering, Georgia Institute of Technology, Atlanta, USA

**Keywords:** Electrical and electronic engineering, Sensors

## Abstract

This paper presents a novel high-Q silicon distributed Lamé mode resonator (DLR) for VHF timing reference applications. The DLR employs the nature of shear wave propagation to enable a cascade of small square Lamé modes in beam or frame configurations with increased transduction area. Combined with high efficiency nano-gap capacitive transduction, it enables low motional impedances while scaling the frequency to VHF range. The DLR designs are robust against common process variations and demonstrate high manufacturability across different silicon substrates and process specifications. Fabricated DLRs in beam and frame configurations demonstrate high performance scalability with high Q-factors ranging from 50 to 250 k, motional impedances <1 kΩ, and high-temperature frequency turnover points >90 °C in the VHF range, and are fabricated using a wafer-level-packaged HARPSS process. Packaged devices show excellent robustness against temperature cycling, device thinning, and aging effects, which makes them a great candidate for stable high frequency references in size-sensitive and power-sensitive 5 G and other IoT applications.

## Timing resonators: Distributed Lamé mode Resonators (DLR)

Timing resonators are crucial components in a range of electronic devices, where they provide a time-based reference, commonly used in consumer electronics, automotive and industrial settings. Square Lamé mode resonators enable a silicon-based timing resonator with high Q-factor and high-temperature stability. However, they have certain limitations, primarily at VHF ranges. Now, a team from Georgia Institute of Technology, USA, led by Farrokh Ayazi have designed and fabricated timing resonators that utilize the in-plane shear nature of the Lame mode to create distributed resonance which overcome the limitations of conventional timing resonators. Combined with high efficiency nano-gap capacitive transduction, they enable low motional impedances with increased transduction area, while scaling the frequency to VHF range. These DLR designs are robust against common process variations and demonstrate high manufacturability across different silicon substrates and process specifications.

## Graphical abstract

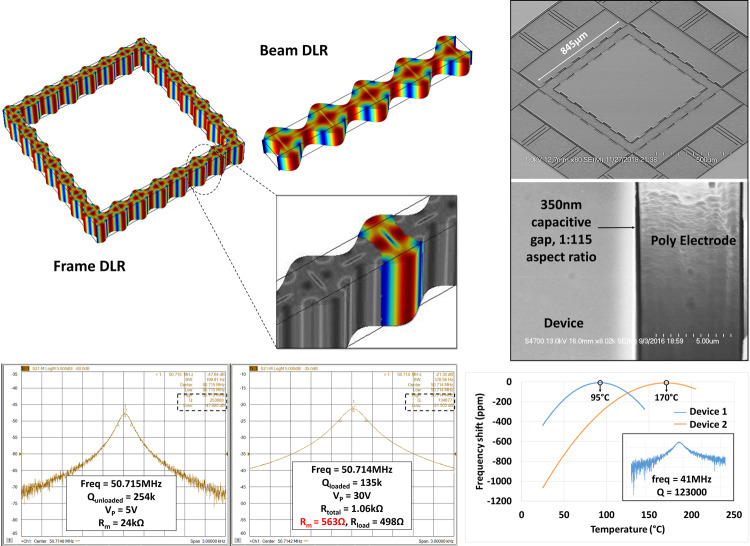

## Introduction

Timing resonators are real-time clocks providing time-base references for electronics in automotive, industrial and consumer applications. Similar to tuning forks used for tuning musical instruments, a high-accuracy timing resonator with high robustness and temperature stability is an essential element for high-performance electronics. For this reason, a substantial part of timing resonator development has been based on quartz resonators owing to their superior temperature properties.

Silicon MEMS timing resonators, on the other hand, have drawn a great amount of attention as an alternative due to their small size, low cost, and more importantly integration compatibility with interface circuits on the same silicon substrate. However, they have had limited success in replacing their quartz counterparts in high-end applications; the bottleneck being the lack of temperature stability and frequency scalability with feasible motional impedance, especially at higher frequencies in the VHF range. In order to overcome the temperature variations, many methods are used^1^, some of which include using compound materials^2^ and using highly doped silicon substrates^3,4^. Using compound materials usually involves complicated fabrications process, leading to higher cost and lower manufacturability. Therefore, the single-material solution is more attractive for reliable mass production. In silicon substrates, the doping changes the relationship of the elastic constants of silicon with respect to temperature, which in turn changes the temperature behavior of the device and improves the temperature stability for certain resonance modes. Specifically, resonators excited into Lamé mode resonance in a highly-doped silicon substrate present a frequency turnover point versus temperature. Square Lamé mode resonators have been popularly used to attain high *f*.*Q* products owing to their low thermoelastic damping (TED) and ability to produce *Q*s over a million with frequencies in the range of 1–10 MHz^3–6^. However, motional impedance requirements for low noise oscillator implementation and mode distortion due to anchoring restrict the minimum feasible size of the square Lamé mode resonators, limiting them to relatively low resonance frequencies that require up-converting frequency synthesizers with to additional phase noise and larger power consumption. Therefore, it is desirable to design a low motional resistance resonator at high frequencies with high-temperature frequency turnover point to overcome these drawbacks and qualify silicon resonators for high-end applications such as high-frequency IoT applications.

Different high frequency resonance modes have been investigated for silicon resonators, however they usually show large linear temperature coefficient of frequency (TCF) and suffer from large temperature instability^7^ in addition to large motional impedance^8,9^. Cross-sectional Lamé mode resonators have been demonstrated previously, eliminating the anchoring limits of square Lamé mode resonators and showing frequency turnover points at high frequencies^10^. However, such designs are sensitive to substrate thickness and have low robustness against process variations, affecting their manufacturability for mass production. Furthermore, their motional impedances can be high in the order of 100 kΩ, adding difficulties in oscillator implementation.

In this work, we present a robust resonator design utilizing the in-plane shear nature of Lamé mode to create distributed resonance, which for the first time, enables high frequency, high-temperature TCF turnover point, low motional impedance, and high manufacturability at the same time. The wafer-level-packaged DLR allows us to achieve high *f*.*Q* products at high frequencies (>50 MHz) in a small footprint and without using getters, which makes it an ideal solution for high frequency, low-power temperature-stable applications. Table S1 in the supplementary information briefly summarizes the enabling features of DLR as compared to other timing BAW resonators. Detailed design, simulation, and experimental results are reported and discussion in the next section, followed by material and fabrication method information.

## Results and discussion

### Distributed Lamé mode design

**Fig. 1:**
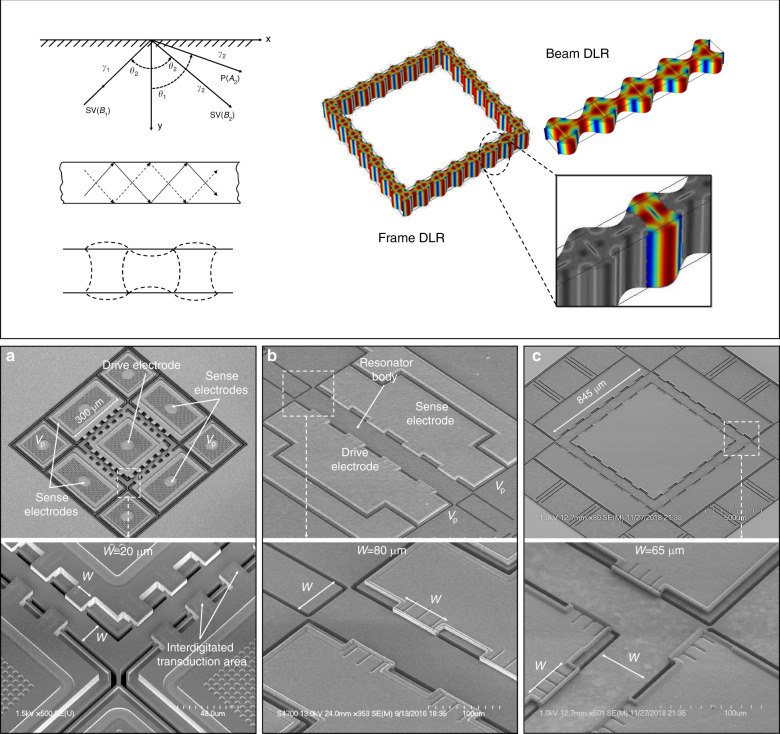
Animation of a propagating train of Lamé modes in the shape of frame and beam structures (top) and (top, inset) highlighted deformation of one distributed element. SEM images of **a** a silicon FDLR, **b** silicon BDLR, and **c** an epi-poly FDLR of various widths. Note that the unique discontinuous interdigitated electrode is required to actuate the distributed mode shapes, which is enabled by the HARPSS process for nano-gaps

The Lamé mode is one of the oldest bulk acoustic modes of vibration with an acoustic formulation^11^, which has been widely used in timing applications because of certain merits such as high Q due to low TED^12^, high temperature turnover points^3,4^ and the simplicity in the mode shape. To scale up the frequency while conserving these merits, it is important to understand the equations that govern traditional square Lamé modes. In typical silicon bulk acoustic wave resonators, primary (P-wave) or secondary vertical (SV-wave) elastic waves traveling through the resonator body reach the silicon-air interface and reflect back to form a combination of P and S waves, a phenomenon also called mode conversion. Now consider an SV-wave with amplitude *B*_1_ incident at the edge of a resonator, which reflects back into a P-wave with amplitude *A*_2_ and an SV-wave of amplitude *B*_2_ as shown in Fig. 1. The ratios of the amplitudes, which are solved using plane wave equations, can be given by ^13^:

1 $$\begin{array}{*{20}{c}} \displaystyle{\frac{{B_2}}{{B_1}} = \frac{{\sin 2\theta _1\sin 2\theta _2 - k^2\cos ^22\theta _2}}{{\sin 2\theta _1\sin 2\theta _2 + k^2\cos ^22\theta _2}}} \\ \displaystyle{\frac{{A_2}}{{B_1}} = \frac{{ - 2k^2\sin 2\theta _2\cos 2\theta _2}}{{\sin 2\theta _1\sin 2\theta _2 + k^2\cos ^22\theta _2}}} \end{array}$$

Here, *θ*_1_ is the angle of reflection of the P-wave and *θ*_2_ is the angle of incidence and reflection of the SV-waves *B*_1_ and *B*_2_ respectively, and *k* is the ratio of the wavenumbers γ_2_/γ_1_. From these equations given in (1), we can calculate that for the special case wherein *θ*_2_ = 45°, we get *A*_2_*/B*_1_ = *0* and *B*_2_*/B*_1_ = *1*. From these two ratios, we can see that for an SV-wave incident at 45°, the resulting reflection is only an SV-wave. This pure SV-wave nature of Lamé mode indicates that a series of Lamé modes can result from a propagating train of S-waves at 45°. Animated representations of a few such modes are shown in Fig. 1. These modes retain the thermal and mechanical properties of a square Lamé mode.

Using the above concept, it is possible to actuate a series of Lamé modes “distributed” in a rectangular beam or a frame with a uniform width of a single square Lamé mode (Fig. 1). Such designs, known as distributed Lamé resonators (DLR) were fabricated on both single-crystalline and poly-crystalline silicon SOI wafers, the process flow of which is explained in the next section. The distributed configuration, especially the frame structure, provides more design freedom in performance scaling. To further explain this, we have to consider the frequency *f* of square Lamé mode resonator aligned to the <100> direction is given by ^14^:

2 $$f = \frac{1}{{\sqrt 2 .W}}.\sqrt {\frac{{c_{11} - c_{12}}}{{2\rho }}}$$

Here, *W* is the width of the resonator, *c*_11_ and *c*_12_ are primary elastic constants, and *ρ* is the density of silicon. We can easily see that in order to get high operational frequency, the width *W* of the square device needs to be extremely small, which significantly sacrifices the transduction efficiency for oscillator applications. The motional impedance *R*_m_ of a square Lamé mode with a pair of actuation and readout electrodes is given by ^15^:

3 $$R_{\mathrm{m}} = \frac{{2\pi \gamma Mfg^4}}{{Q\varepsilon _0^2W^2t^2V_{\mathrm{p}}^2}} \propto \frac{{\rho W^2tfg^4}}{{QV_p^2W^2t}} \propto \frac{{fg^4}}{{QV_{\mathrm{p}}^2}}$$

where *γ* is the mode-shape related transduction coupling ratio, *M* is the effective mass of the resonator, *Q* is the quality factor, *t* is the device thickness, *g* is the transduction gap size, and *V*_p_ is the polarization voltage. For a square Lamé mode to be designed at higher frequencies, we see that the width *W* of the resonator would have to be small which relates to a loss of transduction area, thereby making the motional impedance increasing proportionally with the frequency. In addition, for properly designed Lamé mode resonator, the *Q* is close to the Akheizer limit of silicon, which decreases at high frequencies, further increasing *R*_m_. Consequently, the rapid increase in motional impedance prevents one from up-scaling the operational frequency, which highly limits the application of a square Lamé mode resonator.

In DLRs, length can be extended to a much longer multiple of the width. This does not change the frequency of the device, since the frequency is governed only by the width *W* of the resonator. However, doing this effectively increases the transduction area by allowing a discontinuous electrode arrangement. While the effective mass scales linearly with the number of unit square Lamé mode cells, the combined effect of increased actuation and readout transduction areas scales quadratically as the length of the resonator and number of unit cells increase, thereby improving *R*_m_ and minimizing the insertion loss as compared to a square Lamé mode. For a DLR with 2*N* unit cells and *N* electrode-pair-digits (one electrode-pair-digit for every alternate unit cell), the motional impedance is given by:

4 $$R_{\mathrm{m}} = \frac{{2\pi \gamma Mfg^4}}{{Q\varepsilon _0^2N^2W^2t^2V_{\mathrm{p}}^2}} \propto \frac{{2N\rho W^2tfg^4}}{{N^2QV_{\mathrm{p}}^2W^2t}} \propto \frac{2}{N}\frac{{fg^4}}{{QV_{\mathrm{p}}^2}}$$

Comparing Eqs. 3 and 4 we can see, the increase in frequency and drop in *Q* can be compensated by extending the resonator length and increasing the number of electrode-digits and unit square Lamé mode cells. This shows that the decoupling of width and length offers design freedom to scale the frequency without compromising transduction efficiency; wherein the DLR shows a clear advantage over square Lamé mode resonators.

### Frequency and motional impedance scaling

**Fig. 2:**
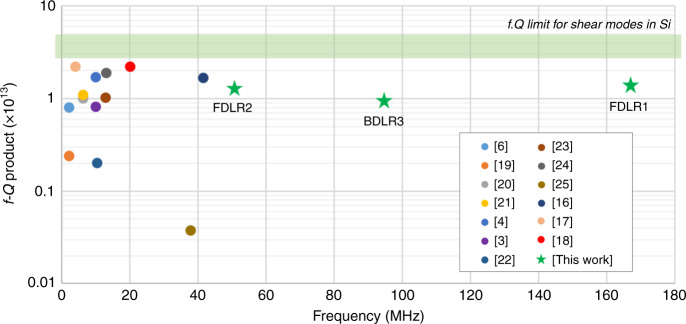
Review of high *f.Q* square Lame mode resonators. Comparison of high *f.Q* in-plane square Lamé modes in literature with the devices designed in this work; a clear distinction can be made in terms of frequency, showing that the DLRs allow us to implement resonators at much higher frequencies than the square Lamé modes, with comparable *f.Q* products and a low motional resistance

To verify frequency scalability and the motional impedance reduction of DLR designs, various DLRs of different widths *W* were fabricated in the beam (BDLR) as well as the frame (FDLR) configuration. The beam or frame width determines the resonator frequency, matching the frequency of a square Lamé resonator with the same width. The various parameters measured for different types of resonators are highlighted in Table 1. Figure 2 shows the comparison of high *f.Q* product in-plane Lamé mode silicon resonators in literature^3,4,6,16–25^ with the resonators fabricated in this work. With the distributed design, the three DLRs with narrow beam width successfully demonstrated high frequencies beyond the reach of any other in-plane Lamé mode designs, while maintaining a high *f.Q* product close to the estimated range of Akhiezer limit for shear modes in silicon^18,26^. More importantly, low motional impedances are achieved on these DLRs despite having much higher frequencies.

**Table 1:** Various beam and frame DLRs of different dimensions, showing the features enabled using this design, with high frequency, low motional impedance, and high *Q*

Design	BDLR1	BDLR2	BDLR3	FDLR1	FDLR2	Epi-poly FDLR
Frequency (MHz)	41	51	95	167	51	58
Width (μm)	80	65	35	20	65	65
Length (μm)	720	845	525	300 × 4	845 × 4	845 × 4
Thickness (μm)	60 ± 1	40 ± 0.5	40 ± 0.5	40 ± 0.5	40 ± 0.5	45 ± 2.5
Gap size (nm)	300	270	270	180	270	340
Electrode-pairs	4	7	8	22	24	24
Q-factor (k)	123	148	94	72	250	86
Rm (kΩ)	80	6.1	19	6.2	0.56	9
Doping level (cm^−3^)	5–7 × 10^19^	1–2 × 10^18^	1–2 × 10^18^	1–2 × 10^18^	1–2 × 10^18^	3–5 × 10^17^

**Fig. 3:**
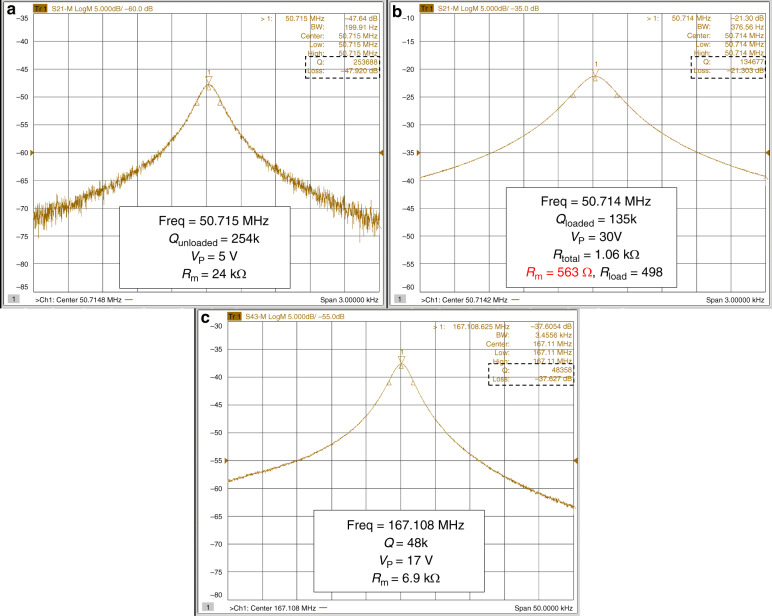
Measured resonance peaks of DLRs. Screenshots of the measured frequency response on a network analyzer of the same FDLR2 device showing the *Q* loading due to its parasitic resistance because of low motional impedance for **a** low and **b** high polarization voltages. **c** FDLR1 measured at 167.1 MHz, showing a high *Q* of 48 k with a low motional impedance

For example, in the FDLR2 version of the resonators, polarization voltage up to 30 V was applied to the resonator body to characterize its motional impedance with a network analyzer (Keysight E5080A). A *Q*-factor loading effect was observe as the polarization voltage increase, which is usually seen for low motional impedance resonators^22^. Measurements with polarization voltage up to 5 V show an unloaded *Q* ~ 254 k, whereas a loaded *Q* ~ 135 k was observed at 30 V. For the loaded Q-factor, we have the following equation ^27^:

5 $$Q_{{\mathrm{loaded}}} = Q_{{\mathrm{unloaded}}} \times \frac{{R_{\mathrm{m}}}}{{R_{{\mathrm{total}}}}}$$

where *R*_total_ = *R*_m_ + *R*_load_, and *R*_load_ is the parasitic resistance in series with the motional resistance *R*_m_ of the device. Based on the insertion loss, the total impedance at 30 V is calculated to be 1.06 kΩ. From Eq. 5 and measured Q-factors, we can calculate the motional impedance of this device at 30 V to be as low as 563 Ω, as shown in Fig. 3. The parasitic resistance *R*_load_ includes the two 50 Ω terminations of the network analyzer, the physical resistance of the resonator body estimated to be 300–400 Ω and small contributions from the through cap vias resistance between 20 and 40 Ω. The existence of body resistance contribution is because for the DLR, the displacement at either side of the electrodes is in phase, as the resonator deforms periodically, net charges will flow in and out the DC port through the body of the resonator, which results in the resistance of the resonator body loading the *Q*^27,28^. This body resistance caused *Q* loading effect has also been shown previously for width extensional SiBAR^29^ and IBAR devices^30^. The simulated values of the *Q*-factors due to different dissipation mechanisms for this design are shown in Table S2 in the supplementary information section. With the Lamé mode having very low thermoelastic damping^12^, it is seen that due to the high-frequency of operation of DLR, the *Q*-factor is limited by a combination of the Akhiezer limit in silicon and the anchor loss. While the *Q*_ANC_ in current designs was ~1 M with 4 µm-wide tethers, simulations show that a modified fabrication process with smaller critical dimension limit will enable *Q*_ANC_ of 80–100 M with narrower or T-shaped tether designs. In addition, incorporating other substrate-decoupling mechanisms such as reflector structures or phononic crystal structures^31–33^ may further improve *Q*_ANC_. The *Q*_TOT_ based on simulations and assumed Akhiezer limit of 2.3 × 10^13^ was calculated to be around 300 k. The slightly lower measured value of 253 k can be attributed to mounting condition or process variation induced anchor loss variation including ±0.5° in-plane crystallographic misalignments (considering commercially available wafers and typical misalignment errors), wherein the *Q*_ANC_ can drop to around 500 k in FEM simulations for the worst case.

A further comparison of reduced motional impedance by frequency scaling can be made from the BDLR3 and FDLR2, which have the same width of 65 µm and thereby the same frequency which is approximately 50.7 MHz as fabricated. With a polarization voltage of 25 V, the loaded *Q*-factor of both devices are similar (~150 k) and the motional impedance are measured to be 1.48 kΩ for the frame DLR and 6.1 kΩ (~4× higher) for the beam DLR, reflecting the 4× more distribution length and number of unit square Lamé mode cells in the frame DLR.

We have previously demonstrated the device FDLR1 with a frequency as high as 167 MHz with a device width of 20 µm on a 40 µm thick substrate^34^, whose motional impedance is only 6.2 kΩ with a relatively low polarization voltage of 17 V which can be generated by typical CMOS circuits. A thick square Lamé mode resonator with such small width and high frequency exceeds the capability of current micro-machining technology for reliable mass fabrication, needless to say the motional impedance of a square device with such dimensions would exceed 100 kΩ, making it improbable for low-noise oscillator implementation. This clearly highlights the advantage that a DLR offers as compared to square Lamé mode resonators. When being used to build oscillators, this low motional impedance and insertion loss will reduce the gain requirement on the amplifier circuits, making it more practical to achieve high oscillation frequencies. Furthermore, for the 167 MHz resonator, an unloaded *Q* of 77 k was measured at 3.5 V^34^, corresponding to an *f.Q* product of 1.3 × 10^13^, which is amongst the highest *f.Q* products measured in silicon.

### Frequency turnover point on highly-doped devices

An important feature of the square Lamé mode is the strong doping dependency of its frequency temperature behavior. A square Lamé mode on a highly n-doped substrate has been known to show a turnover point at high temperatures (>100 °C) for doping level above 4 × 10^19^ cm^−3 ^^35^. At the turnover point, the slope of frequency vs. temperature curve becomes zero. A high-temperature turnover point is important for highly stable oscillator design as it allows for ovenization of the resonator at its turnover temperature to improve frequency stability over the entire temperature range of interest. This important TCF property is maintained for the distributed Lamé mode due to the same nature of wave propagation and energy distribution as a square Lamé mode resonator. The turnover point however, is sensitive to doping variation.

**Fig. 4:**
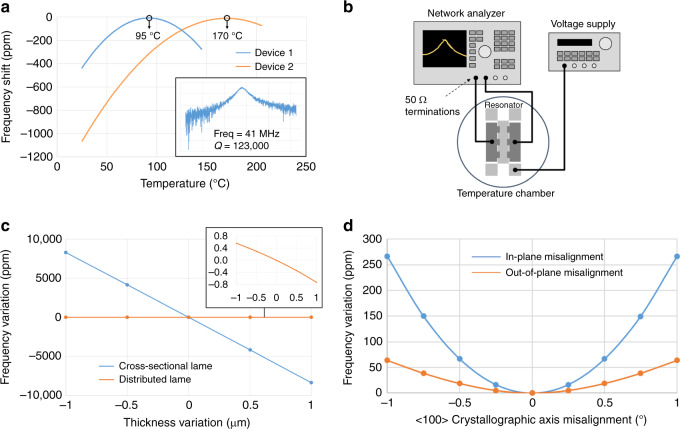
Temperature sweep and crystallorgraphic variations. **a** Measured turnover points at 95 and 170 °C for two beam DLR devices having a doping concentration variation of 5–7 × 10^19^ cm^−3^ and *Q* of 123 k is measured at 41 MHz for the beam DLR1 design (**a**, inset), where the TCF at the two turnover points is zero; **b** measurement setup of the DLR using a temperature chamber and network analyzer for frequency read-out; Simulated **c** thickness variation of ±1 µm shows a large frequency variation of ±8200 ppm for cross-sectional Lamé mode as compared to DLR which shows ±0.6 ppm; **d** variations of ±266 and ±63 ppm for IP and OOP variations respectively in the <100> crystallographic axis

To verify this, the temperature-frequency relationship of the DLR fabricated on highly-doped wafers was characterized in a temperature chamber. This was done on the BDLR1 devices shown in Table 1. A quadratic TCF profile is observed on various BDLR1 devices as expected, with the maximum turnover point measured to be at 170 °C and a minimum turnover point of 95 °C as shown in Fig. 4a, which agrees well with the simulated value for the doping range considering the doping variation across wafers. The TCF around the turnover point is close to zero, therefore ovenizing the device to maintain its temperature at the turnover point will allow one to build an extremely frequency-stable oscillator that are suitable for high-frequency timing applications over a wide range of environment temperature^36^.

### Substrate sensitivity and compatibility

Another advantage of the DLR design is insensitivity to substrate thickness variation and its compatibility with various substrates. One of the major challenges in fabrication of MEMS devices on SOI wafers is the thickness variations on the device layer across the wafer. For commercial SOI wafers with very high doping levels close to the solid solubility of the dopant species in silicon, the thickness variation across the wafer can range from ±1 to ±5 μm.

Previous work^10^ has shown incorporating the Lamé mode in a cross-sectional orientation, enables a high frequency device to be used as a Lamé mode with increased transduction area. However, due to the thickness dependency of the device, the fabrication of such a device becomes difficult and not reliable due to the effect of thickness variation. In contrast, since the DLR is formed by in-plane S-wave reflections, the thickness dependency is eliminated, and better control of the mode shape is enabled even in the presence of large thickness variations as shown in simulated results across thickness variations in Fig. 4c. While a comparable cross-sectional Lamé mode resonator showing ~1.6% frequency variation, the DLR, due to its in-plane nature, has only a frequency variation of ±0.6 ppm over ±1 µm thickness variation.

Another common variation in single-crystal silicon (SCS) substrate is the crystallographic misalignment errors, which were simulated and shown in Fig. 4d. A frequency variation of about 266 ppm was seen for the FDLR2 design for an in-plane crystallographic variation of ±1°, and a 63 ppm variation for an out-of-plane variation of ±1°, which is typical range of errors that can be seen in commercially available SOI wafers. While these frequency variations are tolerable for resonators in SCS substrates, they can be completely negated by using an isotropic substrate such as epipoly-silicon substrate. This highlights one of the advantages of using epipoly as an alternate substrate to fabricate this device, in which crystallographic misalignment errors do not exist due to its isotropic nature. A frame DLR design was successfully demonstrated on an 45 µm epitaxially-grown polysilicon SOI with a thickness variation of ±2.5 µm. Measurements show a frequency of 58 MHz and *Q* of 86 k, with a low motional impedance of 9 kΩ, verifying the compatibility of DLR design with different substrates even with large thickness variations. An epi-poly substrate will also benefit the implementation of high-performance axial symmetric gyroscopes, showing a path towards fully integrated single-substrate timing and inertial measurement unit (TIMU)^37^.

### Manufacturability and reliability

**Fig. 5:**
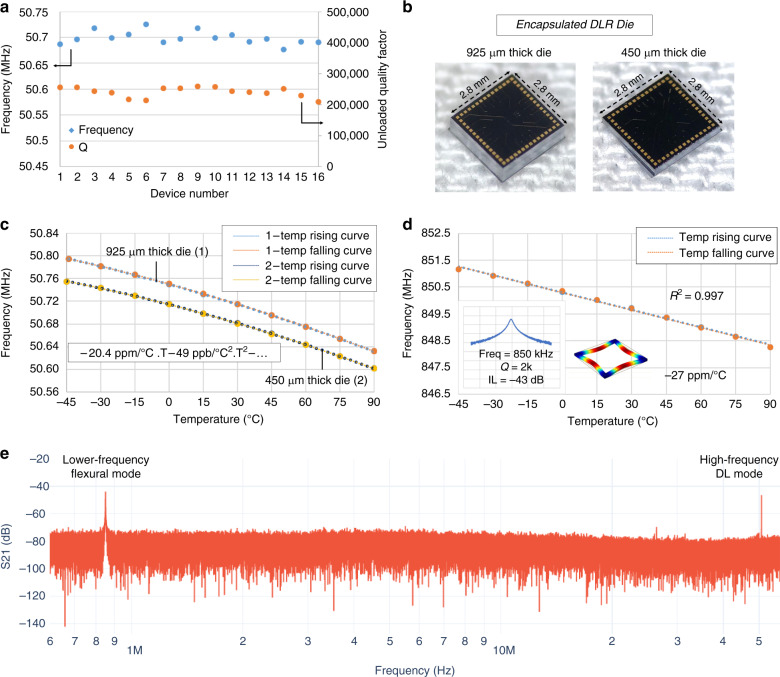
WLP DLR characterization. **a** Measured frequencies and *Q*-factors of 16 WLP die of the FDLR2 design, showing repeatability in yield from device fabrication with as fabricated Δ*f*/*f* spread of 800 ppm within a wafer; the highest *Q*-factor achieved was 253 k from devices close to the center of the wafer; *Q*-factor varies between 200–253 k because of fabrication variations in trench width causing resonator tether widths to change slightly. **b** Optical images of the 925 µm and grinded 450 µm thick WLP dies. **c** Frequency-temperature cycling of both the encapsulated designs, showing negligible hysteresis of <500 Hz between temperature rising and falling cycles with TCF1 and TCF2 of −20.4 ppm/°C and −49 ppb/°C2 respectively. **d** frequency characteristics of the lower frequency flexural mode at 850 kHz showing linear TCF of −27 ppm/°C, which may be used as a reference curve for temperature-stable OCXOs. **e** large frequency span from 600 kHz to 60 MHz, showing no spurious modes for the FDLR2 design

The WLP HARPSS process^37–39^ is an advanced fabrication platform commonly used for creating high-aspect-ratio sub-micron gap devices operating in low-pressure environments. Encapsulated DLR die are fabricated using the HARPSS process and the frequencies and *Q*-factors are measured with good consistency as shown in Fig. 5. Devices across the wafer were tested on three different wafers. Results show a within wafer frequency variation of about 800 ppm at 50.7 MHz, and an across wafers mean frequency variation of 1000 ppm. *Q*-factors were measured to be between 200–253 k with higher Qs measured close to the center of the wafer, which is possibly due to a process variation induced anchor loss difference. In future designs, adding substrate decoupling structures such as phononic crystals to the tether or anchor designs may significantly reduce anchor loss, leaving the DLR Qs limited mostly by the Akhiezer loss in silicon.

Another WLP wafer was grinded down from the backside of the wafer from its original total thickness of 925–450 µm. Figure 5b shows the images of both the original and thinned DLR dies. The DLRs from the thinned wafer were measured and compared with the original device die. Temperature cycling experiments were carried out on both the 925 µm and the 450 µm dies across −45 to 90 °C. Results show that the thinning process did not cause any behavior drift in the resonator with both 925 µm and the 450 µm devices demonstrating consistence repeatable temperature behavior with negligible hysteresis <500 Hz (10 ppm) as shown in Fig. 5c. Both resonators show *Q*-factors of ~200 k with less than 10% variations across the entire temperature range, which verifies the elimination of large TED with strong temperature dependency by conserving the property of undistorted Lamé mode. The thinning experiment verified the robustness of both the DLR design and the WLP process, showing promises for integration of thinned MEMS devices on flexible substrate for advanced wearable applications.

Another important feature for the DLR designs is the presence of a lower frequency flexural mode in the range of 100 s of kHz to 10 s of MHz, according to the length and width of their edge. The flexural modes usually have a linear negative temperature-frequency relationship and a TCF of about −27 ppm/°C, as measured in the FDLR2 design, for which the flexural mode is at 850 kHz with a *Q* of 2 k, as shown in Fig. 5d. This allows us to make use of the linear TCF in the lower frequency mode as a frequency-output temperature sensor in temperature compensation of the higher frequency mode, for example in OCXOs which have been shown previously in literature^3,40^. It is also observed that other than the distributed Lame mode and the low frequency flexural mode, there are no other spurious modes for the frame configuration in the wide range frequency sweep between 600 kHz to 60 MHz, as shown in Fig. 5e, which ensures stable oscillator implementations. The temperature cycling results show that the DLRs may be used as reliable timing elements in high power circuits such as motherboards, where temperatures rise and fall frequently across a wide temperature range.

**Fig. 6:**
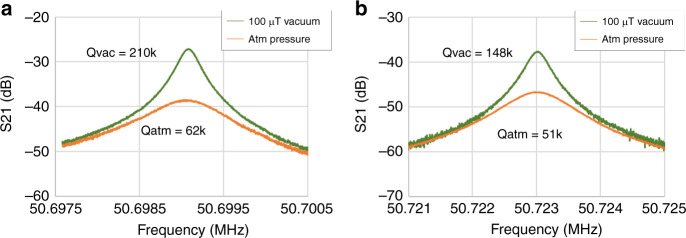
Response of devices in vacuum v/s air. Qs of **a** the frame and **b** beam DLRs of uncapped devices measured in vacuum and air, showing a slight degradation in air due to squeeze-film damping, but maintaining high Qs of 62 and 51k respectively, showing that these devices can also be operated at atmospheric pressure. The degradation of *Q* can be completely removed by designing these devices at a still higher frequency

It is worth noting that owing to their high resonance frequencies, the DLRs do not require very low vacuum packaging below 1 Torr, which would otherwise involve the use of getters and increase process complicity and cost. To verify this, uncapped frame and beam DLRs were tested in both a vacuum chamber with sub-mTorr pressure and in atmosphere. *Q*-factors of 148 and 210 k were measured for the beam and frame DLRs, which matches the typical values seem on WLP dies with 1–10 Torr pressure, indicating the air damping is not a limiting factor even in the WLP dies without getters. It was also seen that the corresponding *Q*-factors are 62 and 51 k in atmosphere, for the uncapped frame and beam DLRs, respectively. While squeeze film damping starts to contribute in atmosphere pressure, the *Q*-factors are still considered to be high enough for timing reference implementation, yielding low motional impedance of 7.8 and 19 kΩ for the frame and beam resonators respectively, as shown in Fig. 6. Compared to conventional lower frequency resonators, which are sensitive to packaging pressure and may fail if the package vacuum is compromised^41^, the high-frequency DLRs demonstrate much better robustness and reliability against extreme environment conditions.

## Materials and methods

The DLRs are fabricated on N-doped SOI wafers using the HARPSS process which enables sub-micron-gap capacitive transduction. 60 μm (±1 μm) and 40 μm (±0.5 μm) SOI were used to fabricate the DLR in beam and frame configurations as reported in the earlier results. A frame DLR was also fabricated on a 45 μm (±2.5 μm) epitaxially-grown polysilicon substrate. It is important to note that the large thickness variation in the epi-poly substrate does not affect the frequency of the frame DLR owing to its robustness to thickness variation. On the SCS substrates, the DLRs were designed to be in the <100> direction, to enable them to show the frequency versus temperature turnover point. It is also seen that rotation in the <100> direction achieves higher *Q*-factors for this mode-shape by lowering anchor-loss in particular.

**Fig. 7:**
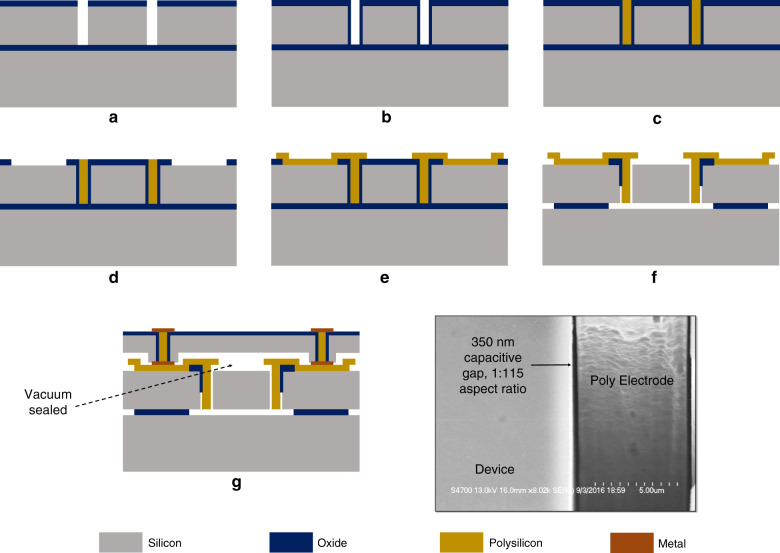
DLR fabrication process. HARPSS Process flow of DLR enabling nano-gaps of 350 nm which result in an aspect ratio of 1:115 for the capacitive gaps (**a**–**g**), with a SEM image of the cross-section of the actual capacitive nano-gap within these devices

The fabrication process flow is shown in Fig. 7. Firstly, trenches are etched in the device layer of an SOI wafer by DRIE using an oxide mask (7a). It must be ensured that the sidewalls of the trenches are as smooth as possible to allow for sub-300nm nano-gaps. Also, it must be ensured that the level of footing inside the trenches is minimal. A thin-film oxidation is then done at 1100 °C (7b). This thin film acts as a sacrificial layer that forms the sub-300nm nano-gap on the sidewall of the trenches. Next, in-situ doped LPCVD polysilicon is deposited at 588 °C in the trenches and then the is etched back to the surface from the top and bottom of the wafer. This is done in multiple steps of deposition and etching in order to mitigate the stress on the wafer, until all the trenches are fully filled (7c). The top oxide is then patterned to open out the electrodes where there needs to be a contact to the silicon device layer (7d). LPCVD polysilicon is then deposited again using the same conditions as step (7b), to form the connections with the silicon and the vertically deposited polysilicon in the trenches (7e). Next, the polysilicon is etched from the trenches other than the electrode regions and finally the device is released in 49% HF (7f). Note that for the epi-polysilicon substrate, the same process is followed, albeit at a reduced temperature to minimize the stress in the released devices^42^. These devices are then capped by eutectic bonding another cap wafer with through silicon vias inside it, providing access to the electrodes at the top of the cap (7g). This bonding process provides 1–10 Torr vacuum of the encapsulated devices.

## Supplementary information

SUPPLEMENTARY MATERIAL
