# Supplementary material for: Low motional impedance distributed Lamé mode resonators for high frequency timing applications
Source: Microsyst Nanoeng. 2020 Jun 15;6:53. doi: 10.1038/s41378-020-0157-z (PMC8433430; doi:10.1038/s41378-020-0157-z)
Supplement: Supplementary file 1 — SUPPLEMENTARY MATERIAL [file 41378_2020_157_MOESM1_ESM.docx]

**LOW MOTIONAL IMPEDANCE DISTRIBUTED LAMÉ MODE RESONATORS FOR HIGH FREQUENCY TIMING APPLICATIONS**

**supplementary material**

Anosh Daruwalla (corresponding author)

adaruwalla3@gatech.edu

School of Electrical and Computer Engineering

Georgia Institute of Technology, USA

Haoran Wen, Ph.D.

haoran.wen@gatech.edu

School of Electrical and Computer Engineering

Georgia Institute of Technology, USA

Chang-Shun Liu, Ph.D.

pantani@gatech.edu

School of Electrical and Computer Engineering

Georgia Institute of Technology, USA

Farrokh Ayazi, Ph.D., IEEE fellow

ayazi@gatech.edu

Ken Byers Professor in Microsystems

School of Electrical and Computer Engineering

Georgia Institute of Technology, USA


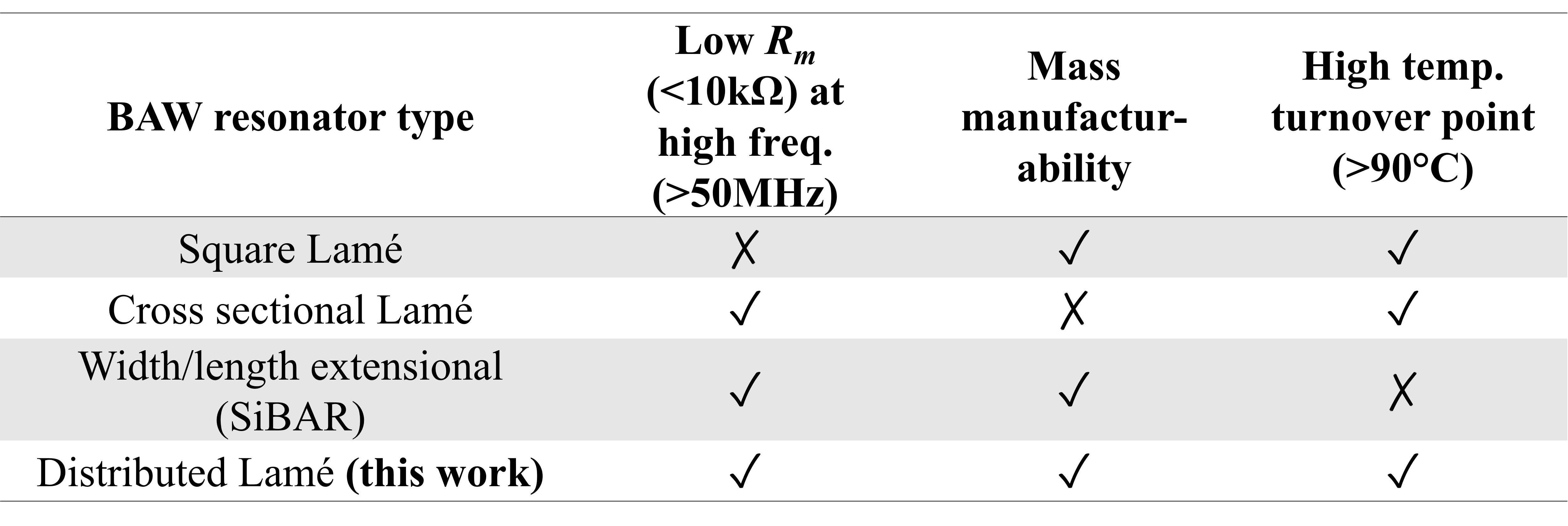


*Table S1: Summary of features enabled by the distributed Lamé mode resonator as compared to other frequently used BAW resonators.*

*Table S2: Simulated values of Q-factors for FDLR2 design. Q_AKH_ calculated based on simulated frequency and a theoretical Akhiezer f.Q product limit of 2.3x10^13^. These devices show a combination of Q_ANC_ and Q_AKH_ in the total Q-facto*
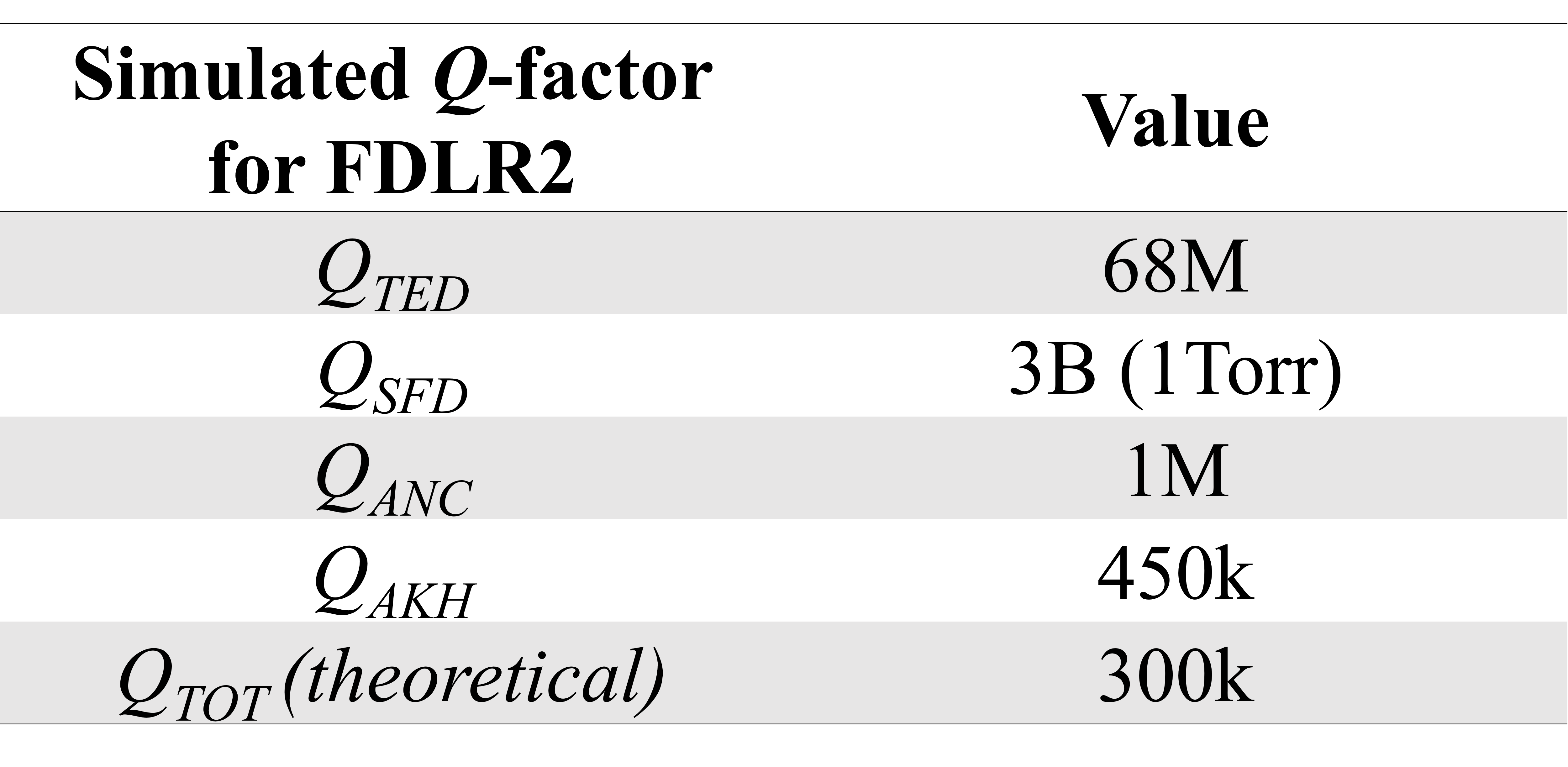
*r. While the Q_ANC_ in this current design was ~1M owing to fabrication limitations, simulations show that a modified fabrication process with smaller critical dimension limit will enable Q_ANC_ of 80-100M with narrower or T-shaped tether designs.*
